# Thyroid Cartilage Metastases from Prostate Cancer on 18F PSMA PET CT: A Case Series

**DOI:** 10.1055/s-0043-1768447

**Published:** 2024-06-26

**Authors:** Poonamjeet Loyal, Samuel Gitau, Khalid Makhdomi

**Affiliations:** 1 Department of Radiology, Aga Khan University Hospital, Nairobi, Kenya; 2 PET/CT Imaging, Department of Radiology, Aga Khan University Hospital, Nairobi, Kenya; 3 Section of Nuclear Medicine, Department of Radiology, Aga Khan University Hospital, Nairobi, Kenya

**Keywords:** thyroid cartilage, prostate cancer, metastases

## Abstract

Prostate cancer metastases to the thyroid cartilage is an extremely rare phenomenon. We report three cases of advanced prostate carcinoma with metastases to the thyroid cartilage identified on 18F prostate-specific membrane antigen-1007 (PSMA-1007) positron emission tomography (PET)/computed tomography (CT). The pathophysiology and possibility for under-reporting are also discussed. Prostate cancer metastases to the larynx should be considered in the differential diagnosis of thyroid cartilage lesions in patients with advanced prostate cancer and is associated with poor prognosis.

## Introduction

Prostate cancer is the second most common cancer diagnosis in men and the fifth leading cause of cancer-related deaths worldwide. 1 Men of African origin have the highest rate of incidence of prostate cancer in the world with incidence of prostate cancer in African Americans almost 60% higher, and the mortality rate two to three times greater than that of Caucasian American men. 2 In Kenya, prostate cancer accounts for 17.3% of all male cancers and 10.2% of all cancers. 3

Skeletal metastases from prostate cancer are a common occurrence seen in approximately 85% of patients dying from prostate cancer and these are predominantly osteoblastic. 4 Metastasis to the thyroid cartilage is extremely rare and here we report three cases of advanced prostate carcinoma with metastases to the thyroid cartilage.

## Case Report

We present three cases of prostate carcinoma seen at our facility for further evaluation and staging with prostate-specific membrane antigen (PSMA) positron emission tomography (PET) imaging.

The first case was a 68-year-old male patient diagnosed with prostate carcinoma in 2016 and had since completed hormonal therapy with radiotherapy. Initial bone scan showed disseminated skeletal metastases. His most recent prostate-specific antigen (PSA) was 99.7 ng/mL. He required restaging examination for evaluation of treatment response. The second patient was an 81-year-old with newly diagnosed prostate adenocarcinoma who presented for staging. The PSA was 160 ng/mL with Gleason score of 4 + 5 = 9.

The third patient was an 88-year-old man who was also diagnosed in 2016 and had completed chemotherapy and radiotherapy in 2017 but was lost to follow-up. His most recent PSA was 3,000 ng/mL.

3D-PET acquisition protocol from the vertex to mid-thighs together with a low-dose CT for attenuation correction and anatomic registration was acquired for all three patients following intravenous administration of 240, 318, and 323MBq of F18-PSMA-1007 with an uptake time of 74, 67, and 69 minutes for the three cases, respectively.

**Fig. 1:**
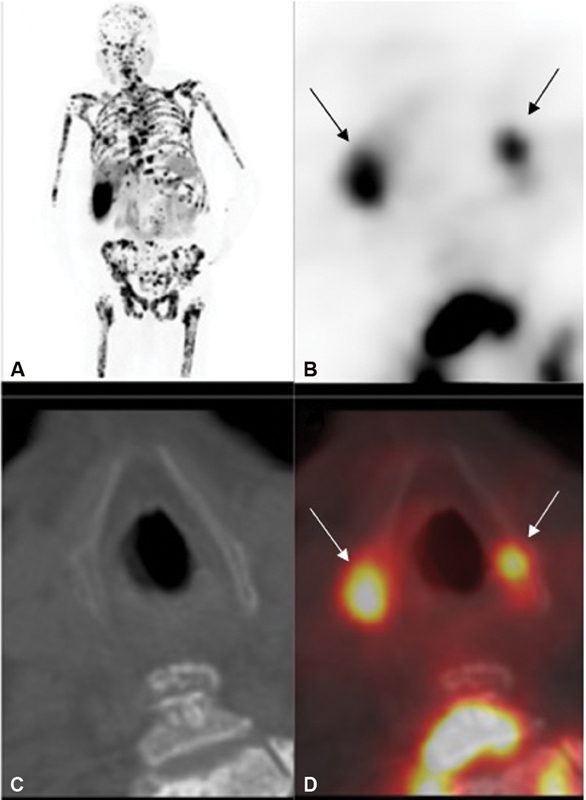
( **A** ) Coronal maximum-intensity projection (MIP) 18F prostate-specific membrane antigen (PSMA) positron emission tomography (PET)/computed tomography (CT) of the first patient shows disseminated PSMA ligand avid skeletal metastases throughout the axial and visualized proximal appendicular skeleton including the calvarium, base of the skull, spine, ribs, sternum, scapulae, pelvic bones, and proximal humeri and femora. ( **B** ) Nonattenuated corrected images. ( **C** ) CT and ( **D** ) axial fused CT images show focal PSMA ligand uptake in the superior cornu of the thyroid cartilage on the right and left.

**Fig. 2:**
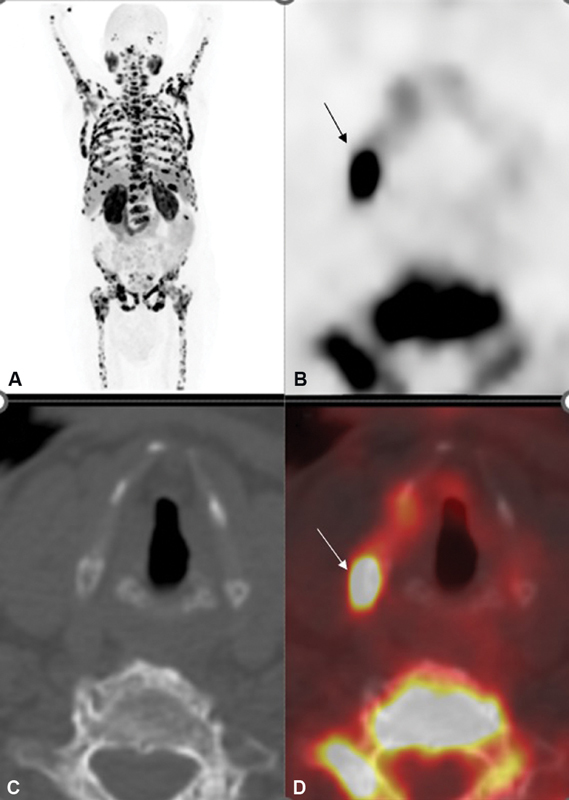
( **A** ) Coronal maximum-intensity projection (MIP) 18F prostate-specific membrane antigen (PSMA) positron emission tomography (PET)/computed tomography (CT) of the second patient shows disseminated PSMA ligand avid skeletal metastases throughout the axial and visualized proximal appendicular skeleton including the calvarium, base of the skull, spine, ribs, sternum, scapulae, pelvic bones, and proximal humeri and femora. ( **B** ) Nonattenuated corrected images. ( **C** ) CT and ( **D** ) axial fused CT images show focal PSMA ligand uptake in the superior cornu of the thyroid cartilage on the right.

**Fig. 3:**
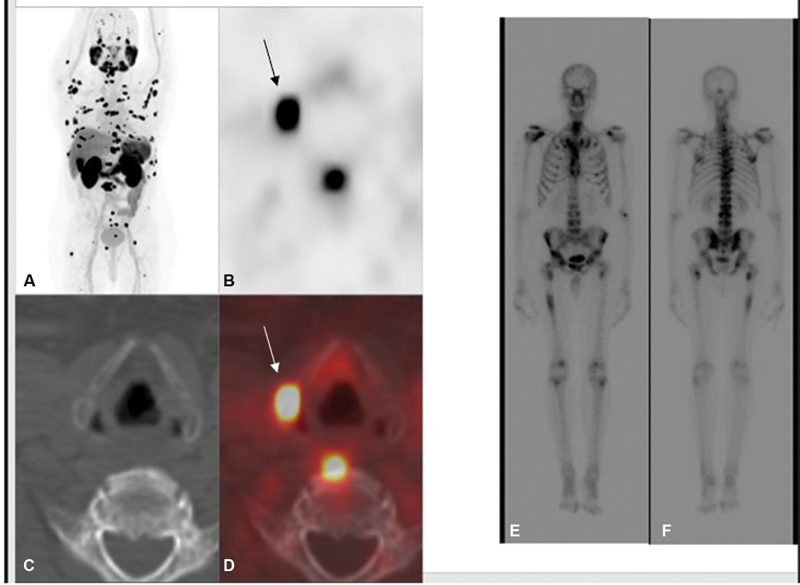
Coronal MIP 18F PSMA PET CT of the third patient ( **A** ), Figure ( **B** ) non-attenuated corrected images, ( **C** ) CT and ( **D** ) axial fused CT images show focal PSMA ligand uptake in the superior cornu of the thyroid cartilage on the right. Bone scan images anterior( **E** ) and posterior ( **F** ) show disseminated PSMA ligand avid skeletal metastases throughout the axial and visualised proximal appendicular skeleton including the calvarium, base of skull, spine, ribs, sternum, scapulae, pelvic bones and proximal humeri and femora.

All three patients had disseminated PSMA ligand avid skeletal metastases throughout the axial and visualized proximal appendicular skeleton including the calvarium, base of the skull, spine, ribs, sternum, scapulae, pelvic bones, and proximal humeri and femora. In addition, there were areas of focal PSMA ligand uptake in the superior cornu of the thyroid cartilage on the right, and the maximum standardized uptake values were 7.6, 23.6, and 34.6 for cases 1, 2, and 3, respectively ( Figs. 1 , 2 , 3 ).

Additional findings for the second patient included increased PSMA expression throughout the prostate gland, with extraprostatic extension but no evidence of PSMA ligand avid disease in the bladder base, seminal vesicles, or rectum. PSMA ligand avid pelvic, retroperitoneal/retrocrural, and thoracic nodal metastases were also seen. There was also nodular bilateral pleural thickening most avid in the left basal pleura, suggestive of pleural metastases. The third patient had a right lower lobe pneumonic process as well.

## Discussion

Most common tumors known to metastasize to the thyroid cartilages include melanoma (29%) followed by renal cell carcinoma (25%) and, to a lesser degree, lung and breast carcinomas. 5 Metastases of prostate carcinoma to the thyroid cartilage is an extremely rare phenomenon and to the best of our knowledge only 14 published cases have been reported since 1908. 6 As in our case, thyroid cartilage metastasis from prostate carcinoma is seen in advanced disease with disseminated skeletal metastatic disease and has been associated with poor prognosis with a survival time of 1 to 3 years. 7 There are also reports in the literature of simultaneous metastases to the thyroid and cricoid cartilage. 8

Metastases to thyroid cartilage is rare due to lack of blood supply within the cartilaginous tissue. The probability of thyroid cartilage metastases increases with age as there is increased ossification of the cartilages. This may be due to metastatic cells being carried in the small thyroid veins and lymphatic vessels, which lead to seeding of micrometastases in the hematopoietic tissue of the ossified segments of the cartilage. 6 These finding are not apparent on conventional imaging at this stage and are only detectable by microscopy. Later these areas lead to destruction, which starts focally and then becomes diffuse with tumor extension into the perilaryngeal soft tissue including the true vocal cords, which eventually leads to dysphonia. 6 At this stage, this gross finding may be identified on conventional imaging.

The second reason for underreporting may be due to reduced sensitivity of bone scan and CT scan, which are the primary modalities used in staging of prostate cancer. The use of the novel tracer PSMA has resulted in increased detection of rare metastatic sites such as in these three cases. Third, even if increased tracer uptake is seen in the region of the thyroid gland on a bone scan, it is often attributed to calcification of the thyroid cartilage and distinction from metastases is difficult.

## Conclusion

Prostate cancer metastases to the larynx are rare but should be considered in the differential diagnosis of thyroid lesions in patients with advanced prostate cancer. They are associated with poor prognosis and can spare the patients further unnecessary workup.
